# Service-Learning Experiences Related to Health Support Activities for Residents Who Have Returned Home after Evacuation Due to a Radiation Disaster

**DOI:** 10.3390/healthcare10081467

**Published:** 2022-08-04

**Authors:** Naoya In, Toshiko Tomisawa, Kasumi Mikami, Mayumi Urushizaka, Kotetsu Tanaka, Chieko Itaki, Maiko Kitajima, Yuka Noto, Ayako Ohgino, Shizuka Takamagi, Kengo Takidai, Chiaki Kitamiya, Yoichiro Hosokawa, Kohsei Kudo, Minoru Osanai

**Affiliations:** 1 Department of Nursing Science, Hirosaki University Graduate School of Health Sciences, 66-1 Hon-cho, Hirosaki 036-8564, Japan; 2 Department of Nursing, Hirosaki University Hospital, 55 Hon-cho, Hirosaki 036-8563, Japan; 3 Department of Radiation Science, Hirosaki University Graduate School of Health Sciences, 66-1 Hon-cho, Hirosaki 036-8564, Japan

**Keywords:** disaster nursing, Great East Japan Earthquake, nursing student, service-learning

## Abstract

Hirosaki University has regularly offered health support activities to residents of X town in Fukushima, and thus, some interactive benefits are expected as a service-learning experience for nursing students. This study aimed to clarify the experiences of students who participated in service-learning and consider which methods and content were effective. In total, 52 nursing students were recruited into the program, which was held from 2018 to 2021. The roles of students included assisting in health consultations related to a radiation disaster. Questionnaires designed by researchers with experience in risk communication programs were conducted on the students after the program, and included the reasons why they joined, their most memorable experiences, and their opinions regarding required support for residents. The data were analyzed by content analysis. The nursing students thought about the health of residents through health support activities in the affected areas. Furthermore, by communicating with residents via on-site service-learning, they could experience the humanity of the residents and the current status of the affected areas, learn the importance of person-to-person relationships, and think about reconstruction. Thus, service-learning was found to be effective and to offer substantial benefits for both residents and students in affected areas.

## 1. Introduction

The Great East Japan Earthquake (GEJE) that occurred on 11 March 2011, had a recorded magnitude of 9.0, making it the largest ever recorded in Japan, and caused a tsunami that resulted in extensive damage to coastal areas. As of 10 March 2021, the number of dead and missing had risen to 15,899 [1], and at 1 week after the disaster, 386,739 evacuees had been displaced [2]. In addition, the tsunami caused an accident at the Fukushima Daiichi Nuclear Power Plant, for which an evacuation zone was established. In X town, Fukushima Prefecture, which is located in the evacuation zone, all 21,000 residents were forced to evacuate to surrounding areas. The evacuation order was lifted on 31 March 2017. As of 31 May 2022, 1878 people, including those returning from evacuation centers, reside in X town in Fukushima Prefecture [3]. At the time of this writing, 11 years have passed since the GEJE. As such, the affected areas have regained their environment and town functions, and residents are starting their new lives in their original area or a new location.

The course of a disaster is divided into five phases as a disaster management cycle, and the current period is considered the “reconstruction phase”. The reconstruction phase is defined as the period of several years after a disaster, during which the affected area gradually begins to recover, such as when evacuees begin moving from evacuation centers into temporary housing. However, during this phase, many victims develop lifestyle-related diseases, chronic diseases, post-traumatic stress disorder, and depressive tendencies, and long-term monitoring is considered necessary [4]. On the other hand, Japan is considered one of the most disaster-prone countries in the world, earthquakes, tsunamis, and typhoons, and one never knows when a disaster may occur. Therefore, in some cases, it is difficult to classify phases, as in the case of the disaster management cycle. On 16 March 2022, an earthquake with a maximum intensity of “scale 6 upper” occurred in Fukushima Prefecture, so that actions are always needed to prepare for the next disaster occurrence.

On the other hand, as of the end of March 2022, nearly 20,000 residents still had not been able to return to X town, and thus continued to live as evacuees inside and outside of the prefecture [3]. The physical and mental effects of the disaster on victims, including health problems caused by radiation exposure, have been reported to be significant [5], as have the effects of temporary housing [6]. Although the time that has passed since the disaster has helped stabilize the mental status of the affected residents, those who have experienced repeated environmental changes, such as living in temporary housing and rebuilding homes, as well as those who have lost family members, are still experiencing mental stress [7]. Therefore, many residents still require additional support to improve their health.

Hirosaki University is engaged in the development of a medical care system, education, research, and human resources related to radiation exposure. In particular, we have been conducting support activities in the affected areas immediately after the accident at the Fukushima Daiichi Nuclear Power Plants. At present, we continue to organize health support activities for the residents of X town in Fukushima Prefecture. On these occasions, we take nursing students to areas where they can provide health support to residents and communicate about the disaster, which brings some interactive benefits. Residents can receive a medical examination, and at the same time, nursing students can gain experience by providing health checks to the evacuated residents and learn more about their current status; this is known as “service-learning”. Service-learning is defined as a “course-based, credit-bearing educational experience in which students (a) participate in an organized service activity that meets identified community needs, and (b) reflect on the service activity in such a way as to gain further understanding of course content, a broader appreciation of the discipline, and an enhanced sense of personal values and civic responsibility” [8]. Service-learning also develops competent health professionals who are responsive to their communities. Service-learning has been widely incorporated into health professional education to foster citizenship and achieve social change [9].

The introduction of service-learning has been recommended in Japanese university education and is expected to have the following effects: 1. Transform the specialized knowledge and skills acquired through specialized education into knowledge and skills that can actually be used in the real world; 2. Provide opportunities to think about future careers; and 3. Improve the qualities and abilities necessary for citizenship by becoming aware of one’s own social role [10].

Various examples of service-learning and its effectiveness in nursing education have also been reported. Outside of Japan, nursing and other health profession students participate in public health emergency training, mass immunizations, vaccination exercises, and outbreak or disease investigations [11,12,13]. In Japan, activities by volunteer groups in university-affiliated districts have been reported [14], as have community health activities in public health nursing practice [15], and the effectiveness of service-learning is becoming evident [16]. However, there are few reports of service-learning for nursing students in the reconstruction phase in areas affected by a radiation disaster, and thus, student learning and the effects of service-learning among nursing students remain unclear. Given this background, this study aimed to clarify the learning and experiences of students who provide health consultations for residents of X town who experienced prolonged evacuation, and to consider the methods and content of effective service-learning.

## 2. Definition of Terms

1. Disaster management cycle

The disaster management cycle is a time-phase regarding the impact of a disaster on individuals, and is expressed as a cycle of hyperacute, acute, subacute, chronic, recovery, and quiet phases, followed by a pre-disaster phase to prepare for the next disaster. Health-care professionals are required to respond to the changing needs of disaster victims based on the disaster management cycle, and to develop nursing care from the perspective of health and livelihoods [4].

2. Reconstruction phase

During the reconstruction phase, people begin to restore lifelines. After the GEJE, even though the evacuation period has been prolonged, in this phase, reconstruction of the affected areas gradually begins as people move from shelters to temporary housing. On the other hand, many victims develop lifestyle-related diseases and experience aggravation of chronic diseases, post-traumatic stress disorder, and depressive tendencies, and these individuals need to be monitored over the long term.

## 3. Materials and Methods

### 3.1. Study Design

Qualitative descriptive research design.

### 3.2. Participants and Recruitment

The study participants were first- through fourth-year nursing students affiliated with Hirosaki University. As mentioned above, Hirosaki University is engaged in education and human resource development related to radiation disaster preparedness. Therefore, students receive basic education on radiation and radiation disaster preparedness from the first year. 

The participants were recruited through a poster and e-mail describing the purpose of the study.

### 3.3. Program

#### 3.3.1. Program Outline

This multi-disciplinary human resource development program was conducted by The Fukushima Innovation Coast Framework, which is a national project designed to build a new industrial infrastructure in the coastal region of Fukushima Prefecture to aid recovery of the industries that were lost as a result of the GEJE, tsunami, and nuclear disaster. The program includes health education, environmental education, and disaster prevention education, and consists of the following three components (Table 1).

#### 3.3.2. Recruitment for programs

Recruitment was performed by sending an e-mail to students explaining the purpose of the program, dates and activities, safety of the area, costs, and other information, as described below.

1. To learn about the current situation in Fukushima and experience reconstruction support in the chronic and recovery/reconstruction phases.
2. To have a valuable experience working in an area where a widespread radiation disaster has occurred.
3. Students should not be concerned about working in X town because radiation levels are at acceptable levels and residents are returning to the area.
4. Dates and activities.
5. All transportation and lodging expenses will be covered.

In addition, the conditions for application were that the applicant must participate in a pre-orientation session about X town and a reflection session after the program, as well as obtaining consent from their family.

#### 3.3.3. Pre-Orientation

Orientation was held for the students prior to their participation in the program. The content consisted of an explanation of activities in X town (e.g., purpose, activities, travel method), viewings of videos showing X town before and after the GEJE (20 min) and residents’ narratives about X town at present (20 min).

#### 3.3.4. Daily Flow

1. Health counseling and risk communication to employees in X town

Medical interviews, measurements of blood pressure, body composition, and vascular age, and health consultations and guidance by medical professionals were conducted for town hall employees at X Town Hall. The students’ roles were to assist with the medical interviews and measurements and to observe the health consultations. The total implementation time was 4 h.

2. Support activities for X town kindergarten and board of education childcare salons

A qualified aromatherapist faculty gave hand massages and made hand cream for mothers and children at a kindergarten in X town. The students’ role was to assist in the making of the hand cream. The total implementation time was 1.5 h.

3. Health consultations and health checks at a rest stop in X town

Nurses conducted a health questionnaire, checked grip strength, body composition, arteriosclerosis, and bone density, and provided feedback to residents at X town’s roadside station. Students assisted in the health consultations and arteriosclerosis and bone density exams. The total implementation time was 3–4 h.

#### 3.3.5. Reflection

After all the sessions were complete, the students and faculty members shared what they had learned. The students presented their experiences on slides and then discussed how to solve problems with the faculty.

### 3.4. Survey Items and Data Collection

We conducted a questionnaire within 1 week after the end of the program using the “SurveyMonkey” online investigation system [17]. The contents of the questionnaire are shown in Table 2.

### 3.5. Statistical Analysis

This study covered the 3-year period of 2018–2021 (Not implemented in 2020 due to COVID-19 infection spread) in the analysis.

The data from the survey responses were repeatedly read by multiple researchers, broken down by contextual meaning and cohesion, and coded at a higher level abstraction. Descriptions were classified and categorized based on similarities and differences in the semantic content of the codes.

### 3.6. Ethics

All study protocols were approved by the Committee for Medical Ethics of the School of Health Sciences, Hirosaki University (2018-018), and informed consent was obtained from each participant prior to the study. There was no disadvantage of students’ score due to non-participation or withdrawal from this study.

## 4. Results

### 4.1. Backgrounds of the Participants

In total, 52 nursing students participated in this program. All students answered all questionnaires (effective response rate: 100%).

### 4.2. Responses to the Questionnaire

The following is a summary of the student responses to each question item. A summary of categories and examples of codes are shown in Table 3. Hereafter, the category name is indicated by double quotation marks (“”) and examples of student responses to the questionnaire are in *italics*.

A total of seven categories regarding the reason for participation were extracted, including “To get experience/learning”, “Because I was interested in something there”, and “To support affected residents”. A total of six categories regarding the most impressive things were extracted, including “Residents’ reactions to health consultations” and “Own activities (Health support activities/Communication with residents)”. A total of six categories of the image before visits were extracted, including “Negative image (e.g., Impact of the earthquake on residents and the environment/Not recovered or in the process of recovering from the earthquake)” and “Positive image (e.g., Reconstruction completed after the earthquake/Reconstruction underway since the earthquake.)”.

Regarding the anxiety before a visit and the reason, 20 students (38.5%) answered “I was not worried at all” and 17 (32.7%) answered “I was not very anxious about visiting there”. The reasons given were “Because I had prior information through news, self-study, classes, orientation, etc.”. Thirteen students (25%) answered “I was a little anxious about visiting there” and two (3.8%) answered “I was very worried about visiting there”. The reasons given included “Because I was worried about health counseling”.

A total of six categories regarding feelings when visiting the town were extracted, including “Reconstruction is underway/Reconstruction was complete (e.g., about new buildings, environments, status of residents, atmosphere)”, “It was under reconstruction/The town has not recovered (e.g., about old buildings, environments, resident status, atmosphere)”, and “Residents have good humanity”.

A total of six categories regarding feelings when communicating with residents and officials were extracted, including “Residents have good humanity/Residents are working hard individually and collectively” and “Residents were affected by the earthquake and radiation”. A total of seven categories regarding important nursing care for residents were extracted, including “Involvement as a health-care professional (e.g., attitude, exploring needs)” and “Anxiety relief/Metal care.” A total of five categories regarding town needs were extracted, including “People interaction/Vitality of people/Creating a place and opportunities for people to communicate” and “Improving the living environment, including facilities and resources/Developing infrastructure”. A total of four categories regarding the things that could be done to help were extracted, including “Engaging with residents through support activities, volunteering, and fieldwork” and “Public relations activities for the affected areas to share with surrounding people and the whole county.” A total of five categories regarding reconstruction were extracted, including “Building a new life and environment” and “Mental changes such as gaining a sense of security and a positive outlook”.

## 5. Discussion

The nursing students who participated in the health support activities in X town, Fukushima Prefecture, answered a questionnaire about what they experienced and what was impressive to them as a reflection after each experience.

Some of the students participated in the program “to support affected residents” or “because I was interested in something there”, for example, volunteering or learning about the lives of residents after the disaster. Many nursing students participated in the program with motivation “to get experience/learning” and expected to experience actual health support activities for residents and learn how to relate to them as health-care providers. Through the activities for the residents, nursing students were impressed by the information and comments about the “residents’ health status and health awareness”, such as “I was surprised at the high bone density of residents in X town”. The nursing students who participated in this program have been learning about the health and nursing care of a target population. It has been reported that compared with the general student population, nursing students are more aware of others (e.g., their attention, concerns, and awareness toward others) [18]. Through communication with the residents, the nursing students naturally became more aware of their health and learned that they lacked knowledge about health as well as places and opportunities for exercise and health activities. Therefore, the present program provided an opportunity for nursing students to consider the health issues of residents in the areas affected by the GEJE.

At the beginning of their activities, the nursing students were busy with unfamiliar health check techniques and procedures. They found themselves unable to communicate with the residents due to nervousness and other factors. However, as the nursing students continued to communicate with residents of various backgrounds and experience health support activities, their communication with the residents improved. The involvement of surrounding people, including other participating students, faculty members who were qualified nurses and health professionals, and residents, had a significant impact on this growth. Subramony [19] reported finding evidence supporting the proposition that both the quality of feedback and students’ dispositions to approach or avoid it positively predict the attainment of learning goals. As nursing students communicated with residents, faculty members monitored student communication and provided advice and feedback as appropriate. Nursing students who received feedback applied it the next time they engaged with residents and as a result, communicated more smoothly; this reaffirmed the importance of faculty involvement. The students followed and imitated the ways senior students and faculty members responded to residents, which contributed to their growth. In his social learning theory, Bandura [20,21] identifies vicarious experience (modeling) and direct experience as factors that increase self-efficacy, with direct experience increasing self-efficacy the most. Reeb [22] also reported that students who engaged in service-learning subsequently had increased self-efficacy. Chen [23] found that the effects of self-efficacy continue across lifespans as the result of accumulated successes and failures in different task domains, and that an increase in an individual’s community service self-efficacy is expected to contribute to an increase in that person’s general self-efficacy. Because we did not measure the students’ self-efficacy in this study, we cannot confirm whether their confidence actually increased, although it can be inferred. Factors that have been reported to enhance the self-efficacy of nursing students include having a good relationship with the subject, receiving praise from others, and acquiring knowledge and social attitudes [24,25]. In addition, behavior change has been shown to be promoted by manipulations that increase self-efficacy [26]. Students mentioned “Residents’ reactions to health consultations” that they found memorable while providing health support activities, including “During the activities, many residents said to me in support, ‘Good luck with your studies,’ which cheered up those of us who were organizing the event and Residents thanked me”. In the course of conducting health support activities in this service-learning program, nursing students were able to build relationships with residents, experience positive changes in their physical and social health, receive direct and indirect appreciation, and gain knowledge and experience. Service-learning was therefore considered to be a learning experience that brought about positive change in various behaviors, such as nursing students’ learning to improve their knowledge and skills as health-care professionals, as well as actions to support disaster-affected areas.

In addition, going directly to the site to see and experience it with one’s own eyes gave the students an opportunity to change their image and impressions. Although the nursing students had watched a video and researched information about the town prior to their visit, more than half still had a “Negative image (e.g., Impact of the earthquake on residents and the environment/Not recovered or in the process of recovering from the earthquake)” such as “It will be deserted there or I think it’s an empty town” beforehand. When the nursing students actually visited and saw the town, they felt that some parts of the town had not yet been reconstructed, and many thought about the residents’ lives in the midst of reconstruction, such as having the impression that “Major home centers and other stores were closed, and life may not yet be the same as before the disaster”, or changed their impression to a different one, such as “I felt that the town was brighter with streetlights and more natural than I had heard, and although there were few buildings and people, I realized that the living environment in X town is being improved”. These realizations led to the awareness that it is important to “Improve the living environment, including facilities and resources/Develop infrastructure”. That is to say, the fact that they actually visited the affected areas, saw the environment in detail, and felt the atmosphere led to concrete learning. There were also many findings gained from interactions with residents in regard to the support and reconstruction needed in the town. By listening to the residents living with the possibility of radiation exposure, they felt that it was important to “provide information to residents” about radiation, and by listening to the residents who wanted more human connection and vitality, they thought that reconstruction also means “Mental changes such as gaining a sense of security and a positive outlook”, “Returning life and the environment to their original state”, and “Vibrancy of people and places/Smiles of the people”. The definition of ‘reconstruction’ is still a topic of debate. Nagamatsu et al. [27] reviewed the concept of reconstruction described in previous studies in regard to the earthquake disaster, and suggested that its definition can be explored by classifying it into the following four approaches: what kind of society do we aim for (philosophical approach), what elements are necessary to achieve reconstruction (mechanistic approach), how to determine and promote reconstruction (governance approach), and what power and technology are needed for reconstruction (capacity approach). The concept of reconstruction discussed by the students overlaps with this approach in many ways, and it was undoubtedly a valuable learning opportunity to get both a bird’s-eye view of the micro and macro perspective.

## 6. Limitation of the Study

This study has some limitations. First of all, it is difficult to generalize the results of this study to all nursing students, because the subjects of this study come from a variety of backgrounds with different grades and experiences. In the future, it will be necessary to examine the differences in learning effects depending on students’ learning backgrounds. In addition, a one-time experience is not sufficient time to learn about the lives of people in the affected areas and their recovery in great details. Repeated learning would further deepen the interactions with the community and its residents. Thus, our future tasks are to analyse the educational effects considering the subject’s readiness and to construct educational programs intended to have repetitive learning effects.

## 7. Conclusions

Nursing students could think about the health of affected residents by visiting the town and conducting health support activities as service-learning. Moreover, they learned many things that could only occur through actual contact with residents and the environment. Through service-learning, the nursing students gained a sense of citizenship and were able to think multilaterally about the reconstruction of the affected areas. Service-learning in areas affected by a disaster during the chronic and recovery/reconstruction phases was found to be effective and provide substantial benefits for not only the residents, but also the students.

## Figures and Tables

**Table 1:** Program list.

Program	Frequency (Times/Year)	Subjects	Contents
Health counseling and risk communication to employees in X town	8	Town Hall employee	Medical interviews/Measurements of blood pressure, body composition, vascular age, etc./Health consultations and guidance by medical professional
Support activities for X town kindergarten and board of education childcare salons	1	Parents and Children	Hand massages and making hand cream by a qualified aromatherapist faculty
Health consultations and health checks at a roadside station in X town	2	Residents	Health consultations/Measurement of blood pressure, body composition, bone density, ABI, PWV, etc.

**Table 2:** Items of the questionnaire.

Items
Which program did you participate in? Why did you decide to participate in this program? How did you feel when you went to the health support activities in X town? What impressed you the most? Did you do any research about X town beforehand? What was your image of X town before you went there? Were you anxious to go to X town? What is the reason? What did you feel when you actually visited X town? What did you feel when you communicate with residents and officials? Would you participate again if you had a similar opportunity? What is the most important care for affected residents? What do you think X town needs now? What can you do to help? What is reconstruction?

**Table 3:** Responses to the questionnaire.

Items	Category (Number of Codes)		Examples
The reason for participation	To get experience/learning (21)		I thought that health consultations could be connected to the work of nurses and public health nurses.
	Because I was interested in something there (15)		I was interested in disaster nursing. I wanted to volunteer.
	To support affected residents (10)		If there was anything I could do to help those affected by the disaster, I wanted to do it.
	Because I’ve never been to a disaster area (9)		I wanted to visit the disaster area in Fukushima once to see what was going on.
	Because it was recommended by others (5)		I wanted to participate after hearing from students who had already participated in the program that they enjoyed communicating with the residents who came for health consultations.
	Because it was a rare opportunity (2)		I thought it would not be easy to experience.
	Because I could go there for free (1)		I was attracted to the free access to Fukushima Prefecture.
The most impressive things	Residents’ reactions to health consultations (20)		During the activities, many residents said to me in support, ‘Good luck with your studies,’ which cheered up those of us who were organizing the event and Residents thanked me. Residents thanked me.
	Own activities (Health support activities/Communication with residents) (12)		I was able to see how to communicate with residents about their concerns and how to build trust with them. Communicating with residents.
	Residents’ health status and health awareness (11)		I was surprised at the high bone density of residents in X town. The residents in X town were trying to understand and learn about radiation on their own.
	Residents’ mental state due to the earthquake (5)		Residents complained that life in temporary housing was stressful.
	Town status (4)		Although 8 years have passed since the earthquake, some banks were not even open, and I felt that the reconstruction of the town had not progressed as much as I had expected.
	Residents’ status (2)		Residents looked no different from the people of their own age who are usually around us.
The image before visits	Negative image (e.g., Impact of the earthquake on residents and the environment/Not recovered or in the process of recovering from the earthquake) (35)		I think it’s all empty lots there. It will be deserted there. / I think it’s an empty town. Reconstruction has not yet progressed there, and there are few residents. It is a town damaged by the earthquake.
	Positive image (e.g., Reconstruction completed after the earthquake/Reconstruction underway since the earthquake.) (11)		I think all the buildings and roads are new. Residents are positive about the reconstruction. Reconstruction is underway there, and people are living there to some extent.
	Image of the earthquake and nuclear power plant (5)		It is a town affected by the Fukushima Daiichi nuclear power plant accident.
	Residents’ lives are not fulfilled (3)		I thought the concerns of the residents must be heavy.
	Ordinary towns (3)		It is a common town. It is a country town.
	Residents spend their time worrying about radiation (2)		I thought the residents were living in fear of radiation.
The anxiety before a visit and the reason	I was not worried at all: 20 (38.5%)		
		Because I had prior information through news, self-study, classes, orientation, etc. (5)	Because I learned about radiation in public health nursing practice.
		Because I understood the safety of radiation (4)	I knew that 10 years had passed, and the radiation levels were up to standards.
		Because I couldn’t think of anything to worry about (4)	I didn’t find anything in particular that made me uneasy.
		I was informed because I or my family is from Tohoku (2)	My father and sister had also visited X town to volunteer, and I had heard about it, so I had no particular concerns.
		Because faculty members were leading (1)	Because I was able to work with the teachers.
	I was not very anxious about visiting there: 17 (32.7%)		
		Because I had prior information through news, self-study, classes, orientation, etc. (9)	I knew it was safe because of the pre-orientation, etc.
		Because I understood the safety of radiation (3)	I looked on the Internet and knew that there was no effect of radiation.
		I was informed because I or my family is from Tohoku (1)	My brother had visited Fukushima for a high school club tournament the year the earthquake happened.
		Because I participated it as a project (1)	Because I will go there as a school project.
	I was a little anxious about visiting there: 13 (25%)		
		Because I was worried about health counseling (5)	I was unsure of how to communicate with residents, and I felt uneasy.
		Because I thought I was affected by radiation (4)	I felt a little uneasy because I could not see the effects of radiation.
		Because it was the first visit to the area (4)	Because I’d never been there before.
		Because I was worried about transportation (1)	I was worried about changing trains, getting to the way stations, etc.
	I was very worried about visiting there: 2 (3.8%)		
		Because I was worried about health counseling (2)	I was worried about whether or not I could do the medical checkups.
Feelings when visiting the town	Reconstruction is underway/Reconstruction was complete (e.g., about new buildings, environments, status of residents, atmosphere) (32)		I felt that the town was brighter with streetlights and more natural than I had heard. Although there were few buildings and people, I realized that the living environment in X town is being improved. It was livelier than I had expected, and I could feel the residents’ desire to make X town more vibrant.
	It was under reconstruction/The town has not recovered (e.g., about old buildings, environments, resident status, atmosphere) (23)		Major home lefts and other stores were closed, and life may not yet be the same as before the disaster. Many large trucks were on the road, and I sensed that the town was still in the process of reconstruction.
	Residents have good humanity (5)		I thought the people of X town were very kind and easy to talk to.
	There was an ordinary country town (3)		There was an ordinary country town.
	There was no radiation concern there (2)		There was some relief for radiation because a radiation monitoring post had been installed at the X town Hall and the numbers were visibly indicated.
	Residents were affected by the earthquake (1)		There are still people suffering from the disaster.
Feelings when communicating with residents and officials	Residents have good humanity/Residents are working hard individually and collectively (18)		I found many of residents to be cheerful and friendly. I felt the community supported each other.
	Residents were affected by the earthquake and radiation (13)		We learned that life in temporary housing after the earthquake was still stressful and difficult. Even after buildings and towns are restored, the memory of the earthquake will remain with residents.
	Residents are in good health and have a high level of health awareness (9)		Residents are interested in their health, have exercise habits and watch their diet.
	Our activities are important (8)		I felt that this is a very good initiative because it provides regular opportunities for such health consultations, which can be a catalyst for behavior change.
	There are no more effects of the earthquake or radiation there (4)		I did not feel any impact from the disaster at all, but rather felt that residents had found enjoyment and were leading fulfilling lives.
	Residents have strong feelings about their community (3)		I felt residents loved X town and were proud of their hometown.
Important nursing care for residents	Involvement as a health-care professional (e.g., attitude, exploring needs) (20)		Listening to the thoughts and feelings of the victims. Accepting feelings as residents are and providing care adapted to their needs rather than actively doing something about them.
	Anxiety relief/Metal care (17)		Mental health care, listening to concerns and worries. To create an environment where residents can feel free to discuss their physical and mental health issues.
	Consideration for the affected residents (8)		As time passed, the emotional scars from the disaster remained, and as an outsider, I felt that we should be cautious about how to close the distance between residents and us.
	Health Support (5)		Providing regular opportunities for health checkups.
	Encourage self-help (5)		Since there are few opportunities to visit a medical institution, guidance should be provided so that they can manage their own health care.
	Providing information to residents (3)		To give correct knowledge about radiation.
	Providing opportunities for communication (2)		I thought it was important to share a good time through events and provide opportunities to engage with various people.
Town needs	People interaction/Vitality of people/Creating a place and opportunities for people to communicate (20)		I think residents need an environment where there are people who listen to them and a community where people living in X town can get together and have fun. I hoped that many young people would move to the area and make the town more vibrant.
	Improving the living environment, including facilities and resources/Developing infrastructure (8)		I thought that gyms, parks, and other facilities where people can exercise were needed to address the lack of exercise, which was one of the most common health concerns. Facilities essential for daily life such as clinics and supermarkets.
	To increase the number of residents and tourists by conducting PR activities for the affected areas throughout Japan (7)		I think it is important for people from other areas to visit X town for sightseeing and events to learn about the current situation.
	Resolving mental and physical problems/Hearing residents’ thoughts/And the place (6)		Decrease residents’ worries and concerns about their lives and health. Support that listens to and accompanies the thoughts and feelings of community members.
	I don’t know/Nothing. (4)		I don’t know because everyone seemed happy and content.
The things that could be done to help	Engaging with residents through support activities, volunteering, and fieldwork (16)		To participate in volunteer and other activities to assist in the reconstruction process. To visit X town and increase opportunities to speak with residents.
	Public relations activities for the affected areas to share with surrounding people and the whole county (13)		I think that by actually going there ourselves and communicating on SNS, it will be one of the opportunities to get people interested.
	Provide health counseling, knowledge, and information as a health care professional (7)		To provide medical support as a healthcare professional. To provide health counseling to residents by acquiring knowledge and skills in radiation, medicine, and health.
	Providing indirect financial support (5)		To buy Fukushima products and donate money from outside.
What is reconstruction?	Building a new life and environment (19)		Setting up the environment in which residents can live happily. Residents choosing new forms of living through disasters.
	Mental changes such as gaining a sense of security and a positive outlook (16)		I think reconstruction is until the residents can live lively every day. I believe it is to be a safe community to live in.
	Returning life and the environment to their original state (15)		It is that the town return to the state it was in before the earthquake happened. To be able to lead the same life as before the disaster.
	Vibrancy of people and places/Smiles of the people (13)		The town is to be a place where many people gather and overflow with vitality. I think it’s about more smiles on the residents’ faces.
	No radiation effects (1)		Being able to stay healthy without the effects of radiation.

## Data Availability

Not applicable.
